# A Multiuser Manufacturing Resource Service Composition Method Based on the Bees Algorithm

**DOI:** 10.1155/2015/780352

**Published:** 2015-08-03

**Authors:** Yongquan Xie, Zude Zhou, Duc Truong Pham, Wenjun Xu, Chunqian Ji

**Affiliations:** ^1^School of Information Engineering, Wuhan University of Technology, Luoshi Road 122, Wuhan 430070, China; ^2^Key Laboratory of Fiber Optic Sensing Technology and Information Processing, Ministry of Education, Wuhan University of Technology, Wuhan 430070, China; ^3^School of Mechanical Engineering, University of Birmingham, Edgbaston, Birmingham B152TT, UK

## Abstract

In order to realize an optimal resource service allocation in current open and service-oriented manufacturing model, multiuser resource service composition (RSC) is modeled as a combinational and constrained multiobjective problem. The model takes into account both subjective and objective quality of service (QoS) properties as representatives to evaluate a solution. The QoS properties aggregation and evaluation techniques are based on existing researches. The basic Bees Algorithm is tailored for finding a near optimal solution to the model, since the basic version is only proposed to find a desired solution in continuous domain and thus not suitable for solving the problem modeled in our study. Particular rules are designed for handling the constraints and finding Pareto optimality. In addition, the established model introduces a trusted service set to each user so that the algorithm could start by searching in the neighbor of more reliable service chains (known as seeds) than those randomly generated. The advantages of these techniques are validated by experiments in terms of success rate, searching speed, ability of avoiding ingenuity, and so forth. The results demonstrate the effectiveness of the proposed method in handling multiuser RSC problems.

## 1. Introduction

Advanced manufacturing technologies or models, such as manufacturing grid (MGrid), global manufacturing (GM), virtual manufacturing (VM), agile manufacturing (AM), and cloud manufacturing (CMfg), have been put forward in order to respond to the rapidly changing market and enhance enterprise competitiveness. The more recent manufacturing models tend to adopt a service-oriented architecture, which can be implemented by using many interoperable standards. This architecture places an emphasis on knowledge and services and aims at realizing full sharing and circulation, high utilization, and on-demand use of various manufacturing resources and capabilities by providing safe, reliable, high quality, cheap, and on-demand used manufacturing services for the whole manufacturing lifecycle [1]. However, in the era of globalization, manufacturing resources are usually characterized by heterogeneously distributed in diverse systems and existed in multiple locations all over the world. Furthermore, these models nowadays incline to accept more open and transparent structures, where a service demander in one system may be a service provider in another system. Therefore the resource service allocation management that is capable of responding to any manufacturing task request rapidly, agilely, and accurately is a critical technique for the current manufacturing models and the next generation.

The shift from production-oriented manufacturing to service-oriented manufacturing necessitates a reform in the methods of resource service composition (RSC). As a key component of resource service allocation management, it takes advantage of existing resource services and integrates them into composite service that can be invoked to fulfill multiple complex manufacturing tasks beyond the capability of any of the services performing singly. The research on RSC in manufacturing environment has increasingly attracted scholars. Some traditional methods for RSC mainly deal with internal manufacturing resources in an enterprise or some in collaborations, or they are unable to handle large-scale RSC optimal selection flexibly and intelligently. Other limitations of traditional RSC methods becoming apparent with the development of new market demands can be reflected as follows. (1) The majority of them strive to accomplish only one task request at a time by providing only one composite service, while ignoring the possibility that multiuser would publish their task requests simultaneously. Consequently the equilibrium allocation of resource services between multiple task requests has been neglected. For instance, resources of high quality may be allocated to a user only because he publishes his task request earlier than others but whose actual requirement is not stringent. (2) Mostly they fail to take into account the diverse situations of the request-and-response manufacturing environment. For example, they may reach a dead end when there are barely sufficient services available or only services of inferior quality available during a certain period but users still launch manufacturing task requests, or they are not competent enough to tackle very demanding task requests for special purposes. (3) Existing researches on RSC have emphasized on trust aggregation and QoS properties evaluation, respectively, for the improvement of RSC, but few of them have integrated the two aspects into one model. In an open manufacturing model a user may get involved in the whole lifecycle of manufacturing; therefore both the subjective properties like trust and objective properties like time and cost play a very important role for the user to make judgment and decisions to select services.

In consideration of the above insufficiencies, this study is to further optimally allocate manufacturing resource service, enhance resource utilization, and promote service composite efficiency. This paper investigates the RSC in a multiuser manufacturing environment, which is a constrained multiobjective combinational optimization problem and has shown to be NP-hard. Both objective and subjective QoS component are involved as objective functions or constraints in the model. The Bees Algorithm as a swarm-based intelligent algorithm is made adapted to solve the problem. Experiments are conducted to look into the performance of the proposed method based on a few criteria such as success rate, fraud service handling, and search speed. How the amended algorithm reacts to the diverse task requests and how its colony size affects the RSC performance are analyzed.

The rest of the paper is organized as follows. The next section introduces the preliminary knowledge and reviews related works on QoS properties evaluation, methods of RSC optimal selection, and the basic Bees Algorithm.  Section 3 proposes a multiuser RSC model based on existing trust aggregation and QoS evaluation methods. This is followed by a detailed description of the Bees Algorithm suitable for solving the problem modeled in Section 4. The representation scheme of a solution, the probabilistic neighborhood search and shrinking strategy, the rules for handling multiple objective functions and constraints, and the time complexity of the algorithm are elaborated. In Section 5, the effectiveness of the proposed method is demonstrated and analyzed by groups of experiments. Finally, Section 6 summarizes the paper and draws directions for further researches.

## 2. Preliminary Knowledge and Related Works

### 2.1. QoS and Trust Aware RSC Optimal Selection

As the number of resource services of similar functionality registered in the service pool increase, users are more concerned about their nonfunctional properties. The functionality refers to what task a service can finish, while the nonfunctional properties are more aware of the QoS such as price, raw material cost, maintainability, and response time. A stand-alone service is often limited in its functionality and a manufacturing task usually demands multiple services in combination as a composite service chain. The technique of evaluating the QoS of a composite service has been investigated for years [2, 3]. Generally, existing researches derive the aggregated QoS from stand-alone services, making the interconnection between them quite significant. The most commonly used composite service structures encompass Sequential, Parallel, Conditional and Loop. The QoS properties are differentiated into three categories: additive, multiplicative, and max-operator. The aggregations of these properties in different categories are given in Table 1 [4]. In the table, *q*(*s*_*i*_) denotes the value of corresponding QoS property of the component service *s*_*i*_. These values should be normalized to the same scale according to Formula (1) in order to avoid inaccuracy and incommensurability due to the distinctive measurement metrics that different QoS properties adopt [5]. In Formula (1), the property value to be maximized is said to be positive, and the one to be minimized is negative. Complicated composite service structures can be simplified by mature techniques, such as transforming to sequential structures [6, 7]. Then the relationship between local and global constraints can be deduced [4]. Apart from this bottom-up method for handling QoS properties, a top-down method is also studied [8], which is claimed to be efficient to address the global constraints, especially for dynamic and adaptable composition of services.

As to the QoS-ware RSC optimal selection, methods and algorithms are constantly being developed. A composition method supporting cross-platform service invocation in cloud environment was studied [9]. The authors put forward a Local Optimization and Enumeration Method (LOEM) for finding a QoS near-to-optimal composite and a decision-making method for supporting cross-platform service invocation in cloud environment. Graph-based service composition was also employed for the purpose of decreasing the complexity of the service composition task [10]. Tao et al. developed a parallel intelligent algorithm named full connection based parallel adaptive chaos optimization with reflex migration (FC-PACO-RM) for RSC optimal selection in CMfg [11]. Recently an increasing number of heuristic searching methods and swarm-based intelligent algorithms are employed to solve the problem, such as the genetic algorithm (GA) [8]. Wu and Zhu introduced an approach that combines the transaction-aware service composition, the QoS-aware service composition, and the ant colony optimization (ACO) together to achieve a trade-off between accuracy and time-efficiency of solving RSC [12]. This algorithm is also exploited for web service composition [13]:

(1) q′si=qsi−qmin⁡siqmax⁡si−qmin⁡si,for  positive  propertiesqmax⁡si−qsiqmax⁡si−qmin⁡si,for  negative  properties.

In addition to the aforementioned RSC methods concerning the objective QoS properties, trust-QoS as a subjective property has also received extensive attention for the reasons presented in previous paragraphs. It is a subjective degree of belief about specific agents [14]. It has been used in proliferation in conducting electronic commerce or business of the Internet in service-oriented environments. In service-oriented architecture, trust is normally divided into four categories: direct experience, trusted third parties, hybrid, and trust negotiation, each of them was described in [15] but the authors did not provide the calculation method. The evaluation models of trust-QoS in MG were proposed [16], in which the trust falls into intradomain and interdomain. The authors also gave a trust-QoS-based MG resource service scheduling framework based on this trust aggregation method. Hammadi et al. [17] used probability theory to determine the trustworthiness of the composite service in the sequential and parallel composite structures. However, probability theory is usually used to determine objective uncertainties and is therefore inappropriate for measuring subjective trust degree. Fuzzy logic and fuzzy number were said to be suitable for modeling the trust in peer-to-peer (P2P) environment, or the trust between users and each business services [18, 19]. The above researches provide the basis of the trust and QoS properties aggregation methods to our model and will not be reiterated in detail.

### 2.2. The Basic Bees Algorithm

The basic Bees Algorithm is a member of the swarm-based algorithm community. It is inspired by foraging behavior of honeybees in nature [20]. A colony of honeybees can extend itself over a long distance and in multiple directions to exploit the most profitable food sources for survival of its colony. During the selection of its nest sites and food resources, they have shown particular characteristics such as precisely navigated, wisely decision-making loop, and well organized. The basic Bees Algorithm is featured by the combination of local and global search, which correlate with the process of exploitation and exploration, respectively. It starts by sending *n* scout bees in the solution space for sampling. These scouts are sorted according to their sampled fitness evaluated by objective functions. The algorithm then selects *m* scouts with better fitness for local search (or neighborhood search) in the neighbor of the scouts. The neighborhood size is specified by *ngh*. Out of the *m* selected scouts, the top ranked *e* (less than *m*) scouts recruit *nre* followers while the rest of the selected scouts recruit *nrb* (less than *nre* due to the lower fitness) followers. During this process, two strategies are employed to enhance the performance [21]. (1) The neighborhood size shrinks if a cycle of local search fails to yield better fitness. (2) A stagnated solution is abandoned to help the algorithm escape from local optima, and the stagnation limit is specified by stlim. Meanwhile, the scouts unqualified for neighborhood search scatter in the solution space randomly again. This step intends to explore unidentified regions that may potentially contain prominent solutions and to maintain the diversity of the scout population. Additionally, predefined stopping criteria are needed to decide when the algorithm should terminate. The Bees Algorithm has been used with great success to calculate almost optimal solutions to a large number of problems like function optimizing and various engineering problems, such as cell formation [22], mechanical design [23], printed-circuit board assembly optimization [24], control systems tuning [25], filter design [26], pattern recognition [27], and chemical engineering [28]. In this paper, the basic Bees Algorithm will be made suitable for solving RSC optimization problem in a multiuser manufacturing environment. Detailed presentation will be provided in the following two sections.

## 3. Formation of the Multiuser RSC Model

A multiuser RSC model is established in this section. Several assumptions should be observed for the establishment. (1) All the manufacturing task requests launched by different users are homogeneous, meaning the requested tasks can be broken down into the same series of subtasks and each subtask shares the candidate service pool individually. (2) The consideration of QoS properties is by no mean exhaustive in this model. Only three QoS properties are exemplified, namely, time, cost, and trust, since the principles of evaluating different aggregated QoS properties have been studied intensively and are analogous in the same composite architecture. (3) All encapsulated stand-alone resource services will not be distinguished from whether they are from the elastic cloud platform or the internet through the outsourcing process.

### 3.1. Denotations

Provided the number of task requests posted at a time is *I*, the request from the user *U*_*i*_ is represented by

(2) $$\begin{array}{c} {\text{Task}}_{i}=\left\{\;\text{s}{\text{t}}_{1},\text{s}{\text{t}}_{2},\ldots ,{\text{st}}_{J}\;\right\}\;\left(i=1,2,\ldots ,I\right), \end{array}$$

where st_*j*_ (*j* = 1,2,…, *J*) denotes a subtask and *J* the number of subtasks. The subtask st_*j*_ can be functionally finished by a service chosen from the service pool:

(3) $$\begin{array}{c} \text{Poo}{\text{l}}_{j}=\left\{\;\text{a}{\text{s}}_{1},\text{a}{\text{s}}_{2},\ldots ,{\text{as}}_{M}\;\right\}, \end{array}$$

where as_*m*_ (*m* = 1,2,…, *M*) represents a candidate stand-alone service (or atom service) in Pool_*j*_ and *M* specifies the number of stand-alone services that Pool_*j*_ contains.

A task request can be responded functionally by a service chain consisting of the selected stand-alone services from each service pool. A service chain can be denoted by

(4) $$\begin{array}{c} \text{Chai}{\text{n}}_{i}=\left\{\;\text{a}{\text{s}}^{1},\text{a}{\text{s}}^{2},\ldots ,{\text{as}}^{J}\;\right\}, \end{array}$$

where as^*j*^ (*j* = 1,2,…, *J*) symbolizes one of the services chosen from Pool_*j*_.

Let *R*_*i*_ be the task request by *U*_*i*_, and it can be expressed as a three-tuple:

(5) $$\begin{array}{c} R_{i}=\langle \;{\text{Task}}_{i}^{r},{\text{time}}_{i}^{r},{\text{cost}}_{i}^{r}\;\rangle , \end{array}$$

where Task_*i*_^*r*^ denotes the user's functional description of the task, while time_*i*_^*r*^ and cost_*i*_^*r*^ stand for the user's QoS requirements for the manufacturing time and cost, respectively. In the model, the users' QoS requirements are scaled into the interval (0,1) and categorized into four degrees that are low: (0.5,1.0), mediate: (0.25,0.75), high: (0.15,0.65), and very high: (0,0.5).

Let *Q*_*i*_ be the QoS evaluation of a composite service, and it can be also represented as a three-tuple:

(6) $$\begin{array}{c} Q_{i}=\langle \;{\text{trust}}_{i},{\text{time}}_{i},{\text{cost}}_{i}\;\rangle , \end{array}$$

where trust_*i*_,  time_*i*_, and cost_*i*_ denote the aggregated trust, time, and cost evaluations of a composite service, respectively.

### 3.2. Trust Management

In this model, trust is managed in two perspectives: (1) direct trust denoted by dTrust_*i*_^(*k*)^(as^*j*^), where *k* counts the number of transactions with the service as^*j*^. It only concerns with those services that the user has directly collaborated with; (2) indirect trust or recommended trust denoted by inTrust_*i*_^(*k*)^(as^*j*^), which is a continuation of direct trust and is more concerned with other users' feedback of direct experience. The method of quantifying the two types of trust is not detailed as there are too many related researches and beyond the scope of this paper. The aggregated trust is calculated as

(7) Trustikasj=α·dTrustikasj+1−α·inTrustikasj,dTrustikasj≠0inTrustikasj,dTrustikasj=0,

where *α* signifies the user's inclination to depend more on self-experience, so *α* satisfies 0.5 < *α* < 1.

A trusted service set (TSS) is introduced to each user, which is expressed as

(8) $$\begin{array}{c} \text{TS}{\text{S}}_{i}=\left\{\;\text{a}{\text{s}}_{l}\;|\;l\leq L\;\right\}, \end{array}$$

where as_*l*_ symbolizes a trusted services belonging to the user *U*_*i*_ and *L* symbolizes the maximum trusted services a user is allowed to have. A stand-alone service is enrolled into TSS only if the number of satisfactory transaction with it in a certain period exceeds a predefined threshold. The next time when the user publishes a task request, the services in the TSS take a priority of being chosen. The TSS is made to be a sequence. It is updated dynamically by the sequence tail (the earliest enrolled trusted service) being discarded and the latest qualified one being inserted into the sequence head. This technique attempts to imitate people's social behavior that they are more likely to socialize with those who they are recently made acquainted and trusted.

### 3.3. Objectives of the Model

Three service composite modes are considered in the model, namely, one-to-one mapping (O2O), many-to-one mapping (M2O), and many-to-many mapping (M2M). Each service chain fulfills only one and at least one task in the O2O mode. This mode is applied to when there are plenty of resource services available. The mode of M2O fits into the condition that users post tough time requirements, so several service chains are allowed to complete one task in cooperation. And the M2M mode is applicable when the resource services become very scarce and it is barely possible to allocate at least one service chain to every task separately. In this mode a resource service can be shared among different users to tradeoff the resource scarcity.

The objectives of the model are established as follows:

(9) max⁡ ∑i=1ITrustik

(10) max⁡ ∑i=1Ixi

(11) s.t. timei<timeir,i=1,2,…,I

The objective (9) realizes the maximization of the aggregated trust that all users hold to the composite service. In the objective (10), *x*_*i*_ is a decision variable. It equals 1 if the task posted by *U*_*i*_ is completed successfully whereas it equals 0 if unsuccessfully. This objective requires the model to satisfy as more users as possible. As to the constraints (11), they point out that the manufacturing time and cost of should be in accordance with users' demands individually. Only when both of them answer to the user's specifications the transaction can be regarded as successful. As can be seen, the established model considers all the users' requirements as a whole to realize a more reasonable resource allocation.

## 4. The Bees Algorithm for the Problem

This section introduces the modified Bees Algorithm as a combinational multiobjective constraint-handling optimizer for the established multiuser RSC model.

### 4.1. Solution Schema

In the modified Bees Algorithm, each scout honeybee represents a solution (a composite service) that answers the calls of users. In the composition mode of O2O each scout can be expressed as

(12) $$\begin{array}{c} S=\left[\begin{array}{c} \left\{\text{a}{\text{s}}_{1}^{1},\text{a}{\text{s}}_{1}^{2},\ldots ,\text{a}{\text{s}}_{1}^{J}\right\}\\ \left\{\text{a}{\text{s}}_{2}^{1},\text{a}{\text{s}}_{2}^{2},\ldots ,\text{a}{\text{s}}_{2}^{J}\right\}\\ \vdots \\ \left\{\text{a}{\text{s}}_{I}^{1},\text{a}{\text{s}}_{I}^{2},\ldots ,\text{a}{\text{s}}_{I}^{J}\right\} \end{array}\right] \end{array}$$

in which the number of service chain is *I* equals the number of users. The stand-alone services composing the solution will be referred as component service in the algorithm. The format of scout in the M2O and M2M modes can be deduced similarly and are not given here.

### 4.2. Scouts Initialization

The initialization process starts by examining whether the user's TSS is empty. If not, a service will be selected randomly from the TSS for building a service chain; otherwise a service will be selected on a random basis from the service pool. In the modes of O2O and M2O, a stand-alone service is not allowed to be shared by several different services chains, meaning an invoked service will not be able to answer the call of other users. However, in the M2M mode, service chain sharing is permitted to compensate for the resource scarcity. Therefore the number of times that a service can be invoked is not restricted. Totally, the number of scout bees to be initialized is *n*.

### 4.3. Waggle Dance

The *n* scout bees are ranked into Pareto dominated sets after being evaluated by objective function and constraints. The purpose is to find Pareto optimality. In Pareto optimality solutions cannot be improved in any of the objectives without degrading at least one of the other objectives. Particular rules are designed as a constraint-handling strategy to sort out the dominated solution sets. In the rules, a feasible solution refers to the one that satisfies all the constraints, whereas an infeasible solution means that it violates at least one constraint. The rules are presented as follows.

1. Any feasible solution shall dominate any infeasible solution.
2. Feasible solutions with better objective fitness shall dominate.
3. For two feasible solutions that have identical objective fitness, the one with larger value for positive properties (or smaller value for negative properties) shall dominate.
4. Among two infeasible solutions, the one violates less constraints shall dominate.
5. Among two infeasible solutions that have identical constraint violations, the one with larger value for positive properties (or smaller value for negative properties) being violated shall dominate.
6. In other cases, the solutions are categorized into the same Pareto set.

After obtaining the Pareto dominated sets, all scout bees are sorted into a sequence according to their dominance relationships. Scouts in the same set are sorted on a random basis. Then, *nre* foraging bees are recruited by the top *e* scout bees and *nrb* foraging bees by the next *m* − *e* scout bees for neighborhood search. The rest of *n* − *m* scout bees are not qualified for recruiting foraging bees and will thus perform random global search.

### 4.4. Neighborhood Search

Probability-based neighborhood search and neighborhood shrinking strategies are developed to facilitate the search in discrete domain. The initial neighborhood size *ngh* is set to be a probability *p*_0_. Each component service in a scout bee correlates with a random number uniformly generated in the interval (0,1). Each foraging bee searches around the scout bee in the form that the component services are replaced by other services in the pool if their corresponding numbers are smaller than *p*_0_, while the others remain as components as before. Afterwards, the dominance between the foraging bee and the scout bee is determined using the rules introduced in Section 4.3. If a foraging bee can dominate the scout bee, it will replace the scout bee and become the new scout in the next search iteration. In the algorithm, this is said improvement has been produced. Otherwise the scout bee retains and the neighborhood size shrinks according to *n*g*h* = *γ* · *n*g*h*, where *γ* is the shrinking coefficient. The failure of producing improvement after a certain number of iterations will force the scout bees abandon its position and reallocate a random one. This step demonstrates the site abandonment strategy in the Bees Algorithm and used when the scout bee is believed to have been trapped in local optima.

### 4.5. Global Search

The remaining *n* − *m* scout bees not qualified for neighborhood search perform the random global search in the solution space. These scout bees are randomized again as they are in the initialization step. This is intended to maintain the diversity of the scout population and explore the solution space to discover potential excellent solutions.

### 4.6. Flowchart and Time Complexity Analysis

**Figure 1:**
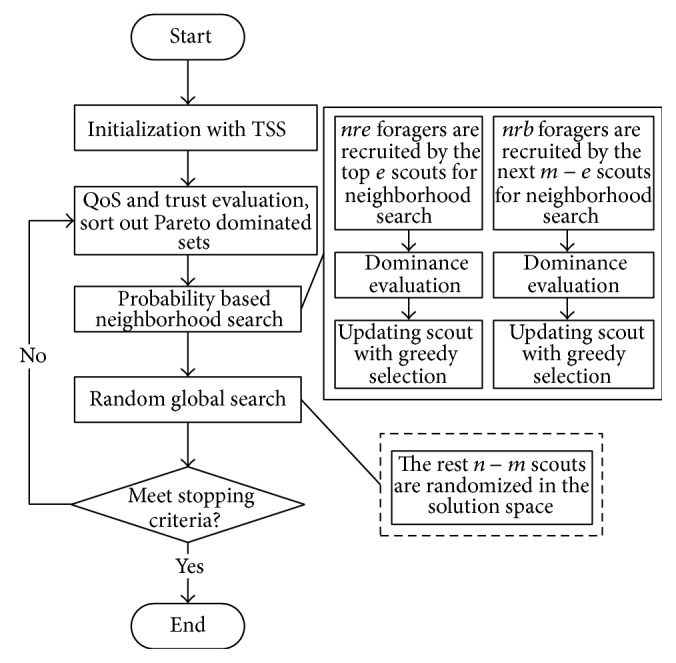
Flowchart of the Bees Algorithm.

The flowchart of the Bees Algorithm for finding an optimal solution to the model is presented by Figure 1. The modified Bees Algorithm uses the parameter nomenclatures stated in previous paragraphs, and the time complexity in one algorithm iteration can be then analyzed as follows:

(1) scout bees initialization: *T*_1_ = *O*(*n* × *I* × *J*),
(2) waggle dance: *T*_2_ = *O*(*n* ^2^),
(3) fitness evaluation: *T*_3_ = *O*(*n* × *I* × *J*),
(4) neighborhood search: *T*_4_ = *O*(*e* × *nre* × *I* × *J* + (*m* − *e*) × *nrb* × *I* × *J*),
(5) global search: *T*_5_ = *O*((*n* − *m*) × *I* × *J*),

of which (2)–(5) are in the main loop. The low power components can be negligible with the scale of the problem increases, provided the number of iteration to is *K*, and the time complexity of the modified Bees Algorithm is

(13) T=Oe×nre+m−e×nre+2n−m×I×J×K.

## 5. Experiments and Discussions

### 5.1. Experimental Setups

Experiments are implemented in three main groups of different complexity under different RSC modes, as shown in Table 2. Groups one and two demonstrate how the composition modes O2O and M2O deal with problems of different complexity. Group three illustrates the way that the mode M2M deals with the situation of insufficient services. For all the experiments, the transaction cycles are set to be 75, and the values of all stand-alone service QoS properties are generated randomly. Moreover, all the users' time and cost requirements fall into their individual range of “low,” “mediate,” “high,” and “very high” on a random basis. For simplification, only the sizes of solution space for O2O in group one and two are calculated, which are 15^7^ × 14^7^ × 13^7^ ≈ 1.13 × 10^24^ and 25^7^ × 24^7^ × 23^7^ ≈ 9.53 × 10^28^, respectively. The modified Bees Algorithm uses the parameter settings as given in Table 3 for different experiment groups unless being specified. Success rate is used as a critical metric to evaluate the effectiveness of the algorithm. It is the number of users whose task requests have been satisfied divided by the total number of users who have published their task requests.

### 5.2. Success Rate

**Figure 2:**
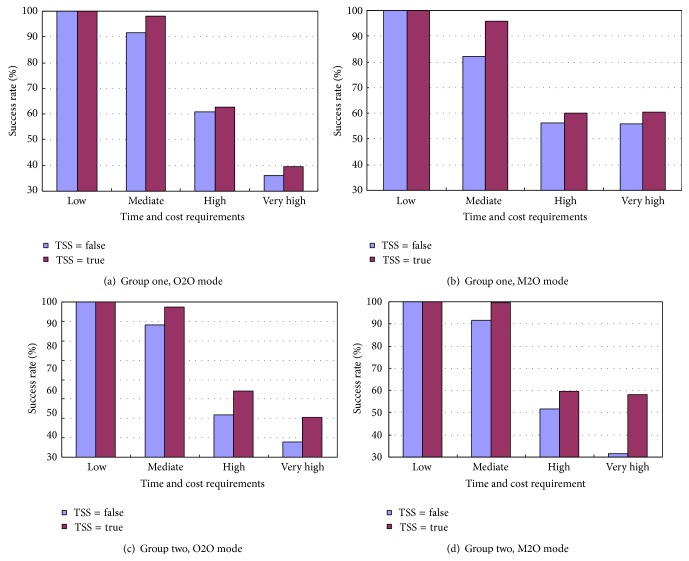
Comparison between the success rates with and without TSS.

The established model introduces the TSS to every user as described previously. Its effectiveness is investigated in the modes of O2O and M2O. The performance of the algorithm using FSS is compared with the algorithm without FSS, as shown in Figure 2. It can be seen that the success rate drops as the users' time and cost requirements become tough simultaneously; meanwhile, the algorithm using TSS outperforms the one without it. This pattern is true for both group one and two. To be specific, the algorithm performs well in dealing with low and mediate requirements (both time and cost), as more than 90% users' requests being satisfied in the mode O2O of group one. Particularly, it reaches 100% success rate when the users have merely low requirements, regardless of the use of USS. However, the success rate drops to around 60% and 38% when the users' requirements become high and very high, respectively. Nevertheless, the algorithm using TSS is able to promote the success rate by approximately 4% on average. The average promotions of success rate are 7.3%, 11.4%, and 11.2% in Figures 2(b), 2(c), and 2(d), respectively. Therefore the utilization of TSS for each user is significant for the algorithm to search for a near optimal composite service at high success rate.

### 5.3. Avoiding Fraud Services

**Figure 3:**
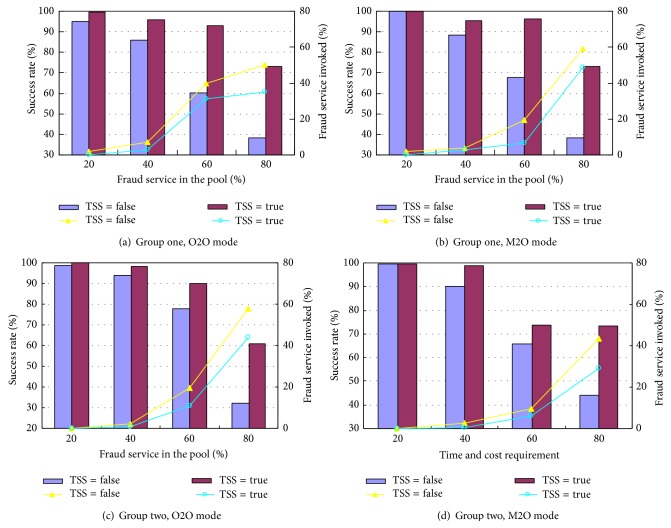
Ability of dealing with fraud services.

The model is capable of preventing user from invoking fraud services to a degree by setting the users' trust as one of the objective functions. The fraud service in the experiment is defined as the service that does not provide genuine QoS information when registering as an encapsulated member of the service pool. It has lower QoS values than it claims but is still capable of completing the task functionally. Both the users' time and cost requirements are kept as mediate throughout this test. The proportions of fraud service in the service pool are set to increase from 20% to 80% with the step of 20%. Details of the result can be referred to Figure 3. In Figures 3(a) and 3(b), which are obtained from group one by the modes O2O and M2O, respectively, the curves imply that an increasing number of fraud services are invoked with the growth of the fraud service proportion in the service pool, and the success rate declines due to this growth. For the algorithm without TSS, only 1.9%, 7.0% and 1.9%, 3.8% of the fraud services have been integrated into the composite service by O2O and M2O, respectively, when there are 20% and 40% fraud services in the service pool, with 95.1%, 85.8% and 100%, 88.4% tasks being successfully fulfilled. As the proportion of the fraud service climbs up to 60% and 80%, 39.6%, 50.0%, and 19.4%, 59.4% fraud services are invoked, respectively, by O2O and M2O, with the corresponding success rates further down to 60.4%, 38.2% and 67.6%, 38.2%. Figures 3(c) and 3(d) display the results from group two, where a similar trend can be seen. However, two vital points can be summarized from the figures that underline the advantages of the use of trust aggregation and TSS: (1) although the invoked fraud service increases with the growth of fraud service percentage in the service pool, the ratio of them being invoked in the composite service is much lower than its actual ratio in the service pool. For example, in the experiment of group two under the O2O mode, the composite service found by the algorithm without TSS contains 0.1%, 2.3%, 19.4%, and 57.8% fraud services when there are actually 20%, 40%, 60%, and 80% fraud service in the pool. It can be calculated that approximately 30% fraud services are avoided on average. (2) The ratio of fraud services included in the composite service can be further reduced if TSS is considered. Again in group two under the O2O mode, the ratio of fraud service being invoked is decreased to 0.1%, 0.9%, 10.8%, and 44.1%, which is further 5.9% lower on average than the algorithm without TSS. Consequently the success rate is raised from 98.7%, 93.8%, 78.0%, and 32.0% to 100%, 98.2%, 90.2%, and 60.9%, respectively, with the growth of fraud service in the pool from 20% to 80%. Hence this experiment indicates that the evaluation of users' aggregated trust can help the users avoid ingenuity to a substantial degree. In fact the use of TSS plays a significant role to further enhance the ability to avoid ingenuity and thus promote the composite success rate.

### 5.4. Composite Speed

**Figure 4:**
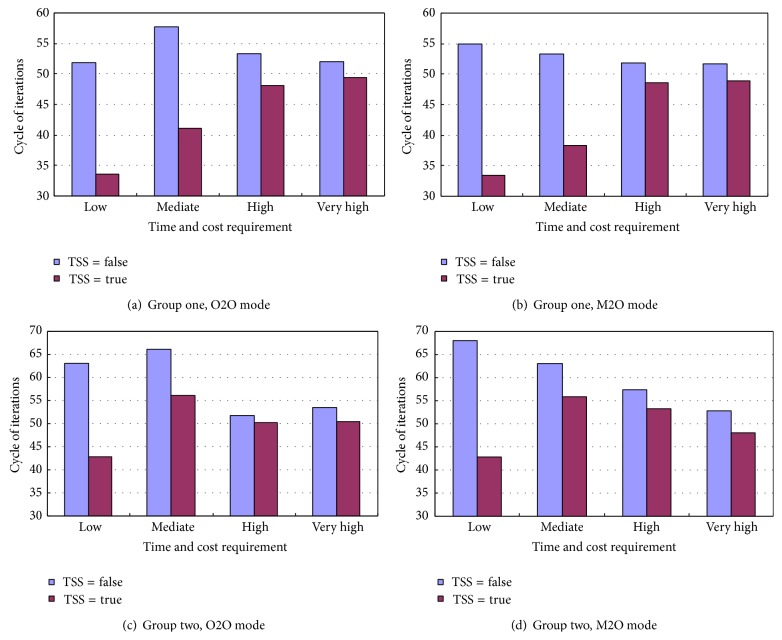
Comparison between the composite speed with and without TSS.

The purpose of this experiment is to investigate the impact of TSS on the speed of searching a near optimal composite service. Also, both the users' time and cost requirements are kept the same in the four degrees. The results obtained from group one and two in the modes O2O and M2O are shown in Figure 4. All the four bar graphs, which represent four varying experimental settings, are characterized by one feature that the bars on the right are lower than those on the left. The feature indicates that the introduction of TSS assists the algorithm in finding an optimal composite faster. The experimental data tells that the TSS helps reduce 18.3, 21.5, 20.2, and 25.2 iteration cycles in dealing with users' low requirements, and 16.7, 14.9, 10.0, and 7.14 in dealing with mediate requirements. The low and mediate requirements are the two situations of which the algorithm can finish the tasks at high success rate. However, it can be observed that the algorithm using TSS does not holds clear advantage over its counterpart when facing the users' high and very high time and cost requirements. As the users' requirements become stringent, the unsuccessful service composition increases. The iteration cycles for an unsuccessful service composition do not make practical sense for statistical calculation; therefore the bars representing the iteration cycles for high and very high requirements do not provide typical characteristic of the speed of successful searches.

### 5.5. Ability of M2O to Handle Various Time Requirements

**Figure 5:**
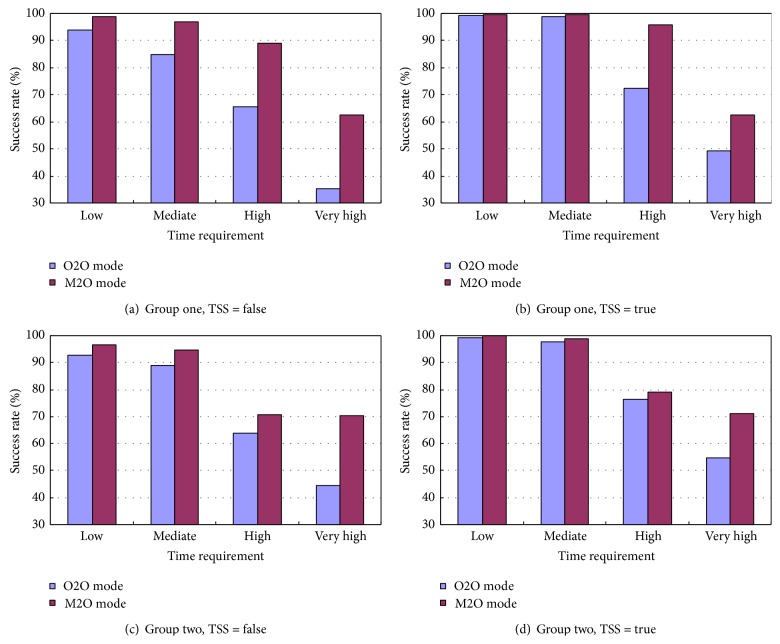
Performance of M2O in handling different time requirements.

The mode M2O is particularly introduced to deal with demanding time requirement. It allows several service chains to work in collaboration for performing one task when it is not possible for one service chain or very hard to find one to complete the required task. This experiment is intended to address the advantage of M2O over O2O in this particular situation. In the experiment, the cost requirement is kept constant as mediate while the time requirement grows from low to very high.  Figure 5 gives the comparison between the performances of the two modes in group one and two. It is understandable that the performances of the two modes in handling low and mediate time requirements do not vary too much, and the use of TSS further reduces this variance. However, the advantage of the M2O mode becomes apparent when the time requirement keeps growing. Take the results presented in Figure 5(b) to demonstrate, if the users only have low or mediate time requirements, the M2O mode fulfills the tasks with 0.5% and 0.7% higher success rate than the O2O mode. However, the M2O mode produces 23.4% and 13.3% higher success rate than the O2O modes in handling high and very high time requirements, respectively, due to the time-saving collaborative working mechanism.

### 5.6. Impact of Colony Size to the Success Rate

Various parameter combinations are used with the aim of investigating how the bee colony size can influence the composite results. Related parameters are listed in Table 4. Some unmentioned parameters like stlim remain their previous values. Both the users' time and cost requirements are kept unchanged to be mediate throughout this experiment.

**Figure 6:**
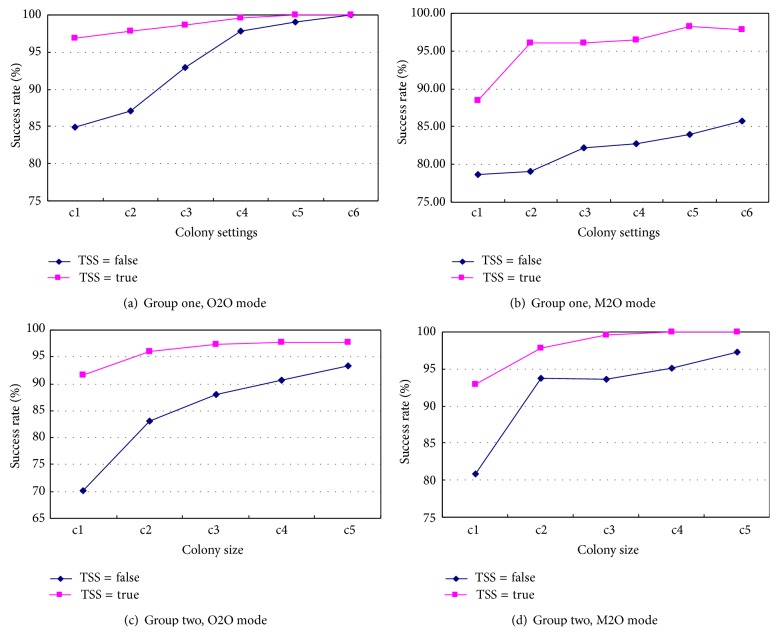
Impact of the colony size on the success rate.

It can be calculated that the colony size grows from the combination c1 to c6 in group one, and c1 to c5 in group two. In this experiment, group one and two do not share a common parameter setting because the RSC problems in the two groups do not have the same complexity. The impact of the colony size on the composite success rate is demonstrated in Figure 6. The curves in the figures imply the tendency of the success rate as the colony size grows. Generally, it can be seen in the four graphs that the increase of colony size enhances the composite success rate. Specifically, noticeable enhancement of the success rate can be observed if the colony size grows from a very small size. However, the enhancement of success rate is not very noticeable if the colony size continues to grow. It is analyzed that the algorithm is able to find a near optimal composite service when its colony grow to certain size as large as c3 or c4 in this experiment. Further augment of the colony will not bring considerable benefit any more. In Figure 6(a), for instance, without using the TSS, the composite success rate climbs up from 84.9% to 87.1%, 92.9% and 97.8% as the colony size grows from 13 to 20, 35, and 70, respectively. The promoted percentage is 2.2%, 5.8%, and 4.9%. When the colony size continues to increase to 105 and 140, the corresponding success rate promotion is down to 1.3% and 0.9%. This pattern can match that in Figures 6(b), 6(c), and 6(d).

### 5.7. Investigation on the M2M Mode

**Figure 7:**
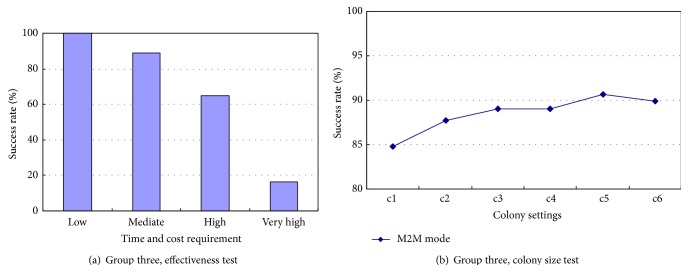
Results obtained in the M2M mode.

The M2M mode is applicable to where many users post task requests but barely sufficient resource services are available. Due to the service insufficiency, the TSS and fraud services are not considered in this experiment in the hope of exploring the potential of all services. Only the effectiveness of the algorithm and the impact of its colony size are investigated. The results are given in Figure 7. It can be seen that the composite success rate can still reach 100% when the users have low time and cost requirements, and this rate remains as high as 89.0% when the users raise their requirements to mediate. The rate continues to decrease to 64.8% and 16.5% as the users post high and very high requirements. A decreasing number of users can be satisfied as they raise their requirement. But the effectiveness of the M2M mode cannot be overlooked because it performs well in solving the problem of insufficient resource service when dealing with low and mediate requirements. In addition, Figure 7(b) tells that the growth of the colony size contributes to the promotion of the composite success rate. However, the enhancement does not endure if the colony size exceeds a certain scale, which resembles the trend in the O2O and M2O modes in the preceding experiments.

## 6. Conclusion

Traditionally, resource service composition methods respond to merely one task request at a time, which could easily lead to misallocation of resources. These methods always search for a composition service of the best quality for one user but ignore a comprehensive QoS trade-off among multiple users. A multiuser RSC method for solving the above problem has been presented and tested on the basis of the established multiuser RSC model. In this model, trust is considered as one of the objectives and QoS requirements as constraints. Particularly, the TSS as a new trust-aware technique is utilized and the experimental results have validated its effectiveness in terms of the enhancement of success rate, the ability to avoid service, and the speed to find a feasible solution. Furthermore, the advantage of the M2O mode in handling tough time requirement and the M2M mode in dealing with resource service insufficiency is also reflected by the results.

The research has not yet been fully completed. The model in this paper does not consider heterogeneous and dynamic task requests. The fault tolerance ability of the model is not investigated either. Furthermore, the investigation on the M2M mode needs to be extended to a more generic study. These problems remain as future research topics.

## Figures and Tables

**Table 1:** The aggregation of QoS properties from composite structures.

Properties	Sequential	Parallel	Conditional	Loop
Additive	∑_*i*=1_^*n*^ *q*(*s*_*i*_)	∑_*i*=1_^*n*^ *q*(*s*_*i*_)	∑_*i*=1_^*n*^ *q*(*s*_*i*_) · *p*_*i*_	*n* · *q*(*s*_*i*_)
Multiplicative	$\prod_{i=1}^{n}q\left(s_{i}\right)$	$\prod_{i=1}^{n}q\left(s_{i}\right)$	∑_*i*=1_^*n*^ *q*(*s*_*i*_) · *p*_*i*_	*q*(*s*_*i*_)^*n*^
Max-operator	∑_*i*=1_^*n*^ *q*(*s*_*i*_)	max⁡{*q*(*s*_*i*_)}	∑_*i*=1_^*n*^ *q*(*s*_*i*_) · *p*_*i*_	*n* · *q*(*s*_*i*_)

**Table 2:** Parameter settings for the RSC model.

Mode	Group one		Group two		Group three
	O2O	M2O	O2O	M2O	M2M
Subtask number	7	7	10	10	7
Service number	15	15	25	25	4
User number	5	5	5	5	7
Request number	3	3	3	3	5

**Table 3:** Parameter settings for the Bees Algorithm.

	Group one	Group two	Group three
*ns *	10	16	10
*nb *	4	4	4
*ne *	1	1	1
*nrb *	5	5	5
*nre *	10	10	10
*stlim *	7	7	7
*ngh *	0.6	0.6	0.6

**Table 4:** Parameter settings for different colony sizes.

	Groups one and three						Group two				
	c1	c2	c3	c4	c5	c6	c1	c2	c3	c4	c5
*ns *	3	5	10	20	30	40	4	10	16	28	40
*nb *	1	2	4	8	12	16	1	4	4	7	10
*ne *	1	1	1	2	3	4	1	1	1	1	2
*nrb *	5	5	5	5	5	5	5	5	5	5	5
*nre *	10	10	10	10	10	10	10	10	10	10	10
Colony size	13	20	35	70	105	140	14	35	41	68	100
